# 

**Published:** 1919-05-31

**Authors:** 


### THE COLLEGE OF NURSING (Limited by Guarantee).

### The London Centre's Club.

THE Club-rooms of the London Centre, at No. 7 Henrietta Street, W. 1, are open to all members of this Centre of the College of Nursing and their friends daily from 10 A.M. to 10 P.M. They are large, comfortable, and quiet. Light refreshments are served from 10 A.M. to 6 P.M. at a very moderate charge. Papers, magazines and stationery are provided. Members have the use of the telephone, and may have letters addressed to the Club. Should they wish letters re-directed, a charge of 5s. per annum will be made. Such letters must be distinctly addressed to the "London Centre's Club," 7 Henrietta Street, W. 1.

Membership of the Club entitles a member to attend all concerts, debates, lectures, excursions, etc., arranged by the Centre, of which due notice is given in the nursing Press. A member of the College of Nursing may become a member of the London Centre's Club by applying to the Secretary. The annual subscription is 5s., payable in April of each year. Any member of the College of Nursing is welcomed as an honorary member in the Club for a period of one month in any year, provided she is introduced by a member of the London Centre.

Members are at liberty to use the Club of any Centre by producing the membership card of their own Centre. They will not be required to pay a further subscription unless they are resident in the district of a Centre for a longer period than six months. In that case members are advised to have their names transferred (through the Secretaries) to the newly selected Centre, and they would be expected to pay the subscription of that Centre.

The Executive Committee welcomes any suggestions for making the Club of interest and help to all members of the nursing profession. It earnestly hopes, when funds allow, to secure attractive residential quarters for its members. The Executive Committee is now arranging for excursions to places of interest during the summer months, and for lectures during the winter session. It asks members to send suggestions, so that the programme may be completed as soon as possible. Notice of arrangements as to time and meeting-place and for the June excursion will appear in the nursing Press of the 13th prox.

### GIFTS AND BEQUESTS.

MR. W. M. BEAUCHAMP, of Limerick, left £500 to the Barrington Hospital in that town.

THE Dowager Lady Jackson has given £1,000 to the Alton Cripples' Hospital to name a cot.

MR. JOHN F. LUND, West Hartlepool, has left £1,000 to the Cameron Hospital, West Hartlepool.

MRS. GERTRUDE MARY TURNER, of Norwich, bequeathed £200 to the Jenny Lind Hospital, Norwich.

THE HON. EMMA MARY MASSEY, of Rodmersham Vicarage, Kent, left £1,000 to the Birmingham and Midland Eye Institution.

A SPECIAL grant of £250 has been made by the Worshipful Company of Goldsmiths to the Great Northern Central Hospital, London.

COL. W. W. CLAPHAM has given a donation of £1,000 to Manchester Royal Infirmary as a "thankoffering for the cessation of hostilities."

THE Westminster Hospital and Charing Cross Hospital each receive £100 from the estate of Miss Ada Marieta Dunn Gardiner, of Chester Square, S.W.

MR. ROBERT HARDIE bequeathed £2,000 each to the Cancer Hospital, Fulham, the Hospital for Consumption and Diseases of the Chest, Brompton Road, and the Royal Hospital for Incurables.

FOR the memorial war hospital for Wrexham and East Derbyshire the trustees of the William and John Jones Hospital Trust have given a site free and are building upon it a hospital at a cost of £50,000.

MISS SARAH CORRY, of St. Leonards-on-Sea, among many charitable bequests, left £4,000 to the National Hospital for Consumption, Newcastle, Co. Wicklow; £1,000 to the Hastings, St. Leonards and East Sussex Hospital; £500 to the Alexandra Home for Chronic Invalids, St. Leonards; £1,000 to the Hospital of St. John and St. Elizabeth, St. John's Wood, as well as the residue of her property.

SIR JAMES INNES, BT., left estate of the value of £45,500. Legacies amounting to about £17,000 were left to friends and relatives, the residue being bequeathed in varying proportions to the following charities:—The Salvation Army; Dr. Barnardo's Homes; St. George's Hospital; Charing Cross Hospital, West London Hospital; Cheyne Hospital for Children, the Samaritan Hospital for Women and Children; and the Cancer Hospital, Fulham.

MRS. ANNIE BURT, of Swanage, left £2,000 to Swanage Cottage Hospital.

MR. ZACHARY SMITH, Derby, left £350 to the Derbyshire Royal Infirmary.

MISS EARLE has made a donation of £500 to the Alton Cripples Hospital and College.

MR. ARTHUR BACKHOUSE, of Torquay, bequeathed £5,000 to Sunderland Royal Infirmary, and £1,000 to Torbay Hospital.

MR. JOSEPH NELSON, of Lancaster, left property of the estimated value of £10,000 for the benefit of the Royal Lancaster Infirmary.

MR. WM. MURRAY, of Leinster Square, W., left £200 each to St. Mary's Hospital and the Paddington Green Children's Hospital.

MISS MABEL RANKEN, of Dewsbury, bequeathed £2,500 to the Dewsbury and District General Infirmary, which institution also participates with two other charitable bodies in the residue of her estate, which is valued at £5,989 net.

MRS. JANET BLACK, of Ayr, Keswick, and London, left £5,000 to the Royal Alexandra Infirmary, Paisley, and £1,000 each to the Keswick Cottage Hospital and Ayr County Hospital.

THE Islington Lodge of Freemasons, No. 1,471, has contributed to the Great Northern Central Hospital a sum of £200 for the naming of two beds for discharged, disabled soldiers and sailors.

THE Association of Infant Welfare and Maternity Centres has received a donation of £1,000, in the form of 5 per cent. War Bonds, from a donor who thinks that the prevention of disease is better than its cure.

MR. W. P. CLARKE, of Regent's Park, N.W., left a fortune of the value of £141,672, which, after the payment of certain bequests, is to be divided between the United Kingdom Beneficent Society, St. George's Hospital, the Lock Hospital, and the National Hospital for the Paralysed and the Epileptic.

CHARING CROSS HOSPITAL receives £1,000 and Brompton and Kensington Hospital £500 under the will of Mr. Joseph Turner, of Earl's Court. His estate is valued at £26,276, and after certain family bequests the residue of the property goes to "such hospitals and institutions as the executors may select, having for their object the benefit of the poor."

### TUBERCULOSIS TOPICS.

### King Edward VII. Sanatorium, Midhurst.

The twelfth annual report has been issued. Three hundred and sixty patients were admitted during the year and forty-eight fewer discharged. A large proportion of the beds have been occupied by patients from H.M. Forces.

A useful feature of the work is a statistical record of all patients passing through the institution since its commencement. By means of this the old sanatorium maxim, that it was of good prognosis for the sputum to become free of bacilli during the patients' stay there, has been confirmed. There is no other special point of interest, although Dr. St. Clair Thomson continues to find galvano-puncture of use in laryngeal tuberculosis. One looks in vain for any mention of treatment by lung collapse. This mode of therapy should have been actively pursued at an institution of this nature, not only now, but five years ago at least. Small countries like Sweden, Denmark, and Switzerland are far ahead here of Great Britain. If we remember rightly, this sanatorium was also slow in adopting tuberculin.

### Newspaper Surgery.

The belief of the man in the street in what he sees in the newspaper is a simple thing, but without it the life of a nation would be profoundly different. Whether life would be worse may be doubted, if we remember the inaccuracy that special knowledge of the subject generally reveals in these modern substitutes for the ancient demagogue. A recent instance is a cutting headed "Knife for Consumption—Danish Professor Claims to Cure Disease by Surgery." It goes on to relate Sangman's having shown a successful case of extra-pleural thoraco-plasty; but evidently considers the thing a discovery. Spengler, Sauerbruch, Wilms, and others, of course, long preceded Sangman, accomplished phthisiologist though he is. The operation is a routine one in Germany and Switzerland. Sometimes, but not often, is the layman introduced by the newspapers of the present day to that which is truly new in the doctors' world—and even then perhaps he were better in ignorance until practical perfection had been more nearly attained.

### Passing Events and Topics.

MR. THOMAS DAVIDSON, 177 West Regent Street, Glasgow, has offered to found and endow a cottage hospital for Girvan. The offer has been gratefully accepted.

EFFORTS are being made to raise £20,000 for the provision of a new wing at the Victoria Central Hospital, Liscard.

IT is proposed to build a cottage hospital for Knutsford, at a cost of £10,000.

WALSALL GENERAL HOSPITAL proposes to provide a fully-equipped pathological laboratory, increased accommodation for the $x$-ray apparatus, a small isolation ward, and a new ward with a minimum accommodation of twenty beds.

IT has been decided at Grimsby to bring the existing hospital buildings up to date and to erect a convalescent home at Humberstone.

### THE COLLEGE OF NURSING, LIMITED.

### Fourth Annual Report, 1919.

The Council of the College, in presenting its fourth annual report, records with great satisfaction the continued confidence in the policy of the College shown by the increased membership, the number of nurses who have joined the College since its establishment, just over three years ago, being 13,014 to the end of the financial year, March 31, 1919. It is also observable with what interest members view the powers afforded them by the democratic constitution of the College and the opportunities of exercising such powers through the postal vote. One-third of the Council having retired last year, the second retirement in rotation takes place this spring, and next year the entire Council will have become elected.

Nomination papers are now in the hands of members, and it is hoped that, in recording their votes, they will endeavour to secure that every branch of the profession is represented on the Council. The amount of time that a candidate for election is able to place at the disposal of the College should also be taken into consideration.

### State Registration of Nurses.

It will be remembered that at the presentation of the last annual report efforts were still being made to arrange for an agreed Bill with the Central Committee for the State Registration of Nurses. This effort was continued until December last, when the Central Committee wrote to the Council saying that "as a result of deliberation the Committee passed a resolution to the effect that its own Bill was a better Bill than that drafted by the College of Nursing, and should be adhered to." The Committee asked the Council to support the Bill framed by the Central Committee as a joint Bill; this the College was not able to do in view of the promise given to its members that any Bill which they supported should provide that two-thirds of the General Nursing Council should be elected by the nurses on the General Register.

Last month the Central Committee was fortunate in securing fourth place in the ballot of Private Members' Bills in the House of Commons, and was thus enabled to introduce its Registration Bill. On the introduction of the Bill, the College Council, being entirely in sympathy with the *principle* of State Registration, supported the second reading, which was carried without a division.

Amendments, however, were drafted on behalf of the College, and an endeavour made to arrive at agreement during the Committee stage of the Bill on the following vital points:

(*a*) Two-thirds of the General Nursing Council to be elected by the women nurses on the General Register.

(*b*) On the Initial Nursing Council equal representation of the College and of the Central Committee.

(*c*) The powers of the Initial Nursing Council to be limited to defining the conditions under which existing nurses may be admitted to the Register.

(*d*) The registration fee not to exceed one guinea.

In the Bill drafted by the College equal representation of the Nurse Societies and of the College was provided on the Initial Nursing Council.

In the Bill of the Central Committee eleven places were appropriated by the Societies (including the Royal British Nurses' Association) affiliated to it, and only four were allotted to the College.

In view of the large membership of the College, and the impossibility of obtaining reliable information as to the number of fully-trained nurses in the other societies, and of their representative character, this inequality could not be accepted.

The Bill is still being discussed in Parliament, and the representatives of the College are fighting for an equitable measure.

### Headquarters of the College.

The question of accommodation for offices of the College was considered early in the autumn of last year, the premises at Nos. 3 and 6 Vere Street proving inadequate to meet its growing needs. Endeavours were made to find a building in the vicinity of the late office, and the Council was fortunate in securing No. 7 Henrietta Street, Cavendish Square, W. 1, which occupies a most central position, and is, moreover, of such character as to meet admirably the needs of the College. After the completion of the renovation of the building the office moved into it last January. The first floor of the building is rented from the Council by the London Local Centre, and is used as club-rooms for its members. The Centre held a reception on the day of the opening, and many nurses and friends of the College were present.

Members of the College will ever be grateful to Sir James Boyton, who has proved a valued friend in accommodating the College for nearly three years at No. 6 Vere Street, free of all outgoings, and, although the comfort of the new offices cannot be overestimated, members will always look back with affection to the building where the College was established.

At headquarters is the committee-room where the Council and its committees meet to transact the business of the College, whilst the rest of the building is occupied by offices of the different departments.

### The Nation's Fund for Nurses.

The Nation's Fund for Nurses has a twofold aim, namely: (*a*) To raise an endowment fund for the College; (*b*) to form a tribute fund for the benefit of all nurses, whether members of the College or not, who have been broken either in the war or in the service of the sick. This Fund, so successfully organised by the British Women's Hospital Committee and Miss C. May Beeman, has assisted a great deal towards the endowment of the College, and has made it possible for it to offer studentships to its members.

The studentships offered for the purpose of qualifying members as sister tutors were held at the Household and Social Department, King's College for Women, University of London. An entrance examination for these studentships took place in August of last year, and twelve students entered, the three following being successful: Miss Dorothy E. Bannon, trained at St. Thomas's Hospital; Miss Dianah Marion Edgell, trained at St. Thomas's Hospital; Miss Madge Elizabeth Abram, trained at the Royal Infirmary, Manchester. The Selection Committee, in awarding the studentships, paid special attention to the past experience and references of the candidates, and the total number of marks gained in the qualifying examination was only one factor in the selection of the successful competitor.

These three students took up their residence at King's College for Women in October last. The Council regrets to record that one of the most promising of them, Miss D. M. Edgell, died of pneumonia in November. The other two members conclude their studies in June next.

The College is making arrangements for similar studentships for sister tutors in the autumn.

### The Tribute Fund.

The Tribute Fund has done fine work during the last few months, in a field which was peculiarly untouched in the past, namely, the expression of gratitude by deeds, not words, to the nursing profession. All cases of hardship and need among nurses receive sympathetic consideration by the committee of the Fund, and already a great many grants of money, for weekly sick pay, convalescent homes, sanatorium treatment, have been arranged to help various cases.

**The College of Nursing Report**—(*continued*).

The following extract from the letter of a nurse who had received some financial assistance before leaving England for one of our Colonies is only one of many received at the office: "I thank you for your very kind letter, which brought me great relief of mind. I believe the Tribute Fund is very little known among nurses generally. I much appreciate your interest, and very helpful advice, and shall always retain short but happy associations in my mind of the College of Nursing."

LOCAL CENTRES.

Nurses are becoming more and more awake to the weight of the College and its power for good among them. A wonderful increase of enthusiasm and interest is shown in the desire throughout the country for the formation of fresh local centres.

Nineteen of these are already established, and many invitations have been received by the organising secretary to inaugurate fresh centres. Post-graduate lectures, social evenings, country rambles, *fêtes*, and concerts have been the means of drawing many nurses together and encouraging interchange of thought. Many of the centres are on a sound financial footing, and have been able to secure club-rooms for their headquarters. Those less fortunate have, nevertheless, been able to enjoy the hospitality of a hospital board-room or other meeting place, kindly provided by the local authorities.

The following is a list of the local centres, with the addresses of the honorary secretaries: ABERDEEN.—Miss Edmondson, R.R.C., Royal Infirmary, Aberdeen. BIRMINGHAM.—Miss Colburn, Q.V.J.N.I., 94 Moseley Road, Birmingham. BRIGHTON.—Miss A. M. Latham, Royal Sussex County Hospital, Brighton. BRISTOL.—Miss McHardy, Chesterfield Nursing Home, Clifton, Bristol. CAMBRIDGE.—Mrs. Loughman, Sister Addenbrooke's Hospital, Cambridge. DERBY.—Miss Haines, Children's Hospital, Derby. EDINBURGH.—(1) Miss Turnbull, Matron, Deaconess Hospital, Edinburgh: (2) Miss Cathcart, The Elms, Whitehouse Loan, Edinburgh. GLASGOW.—Miss Lindsay, Matron, Belvidere Fever Hospital, Glasgow. LEICESTER.—Miss Masters, Poor-Law Infirmary, Swain Street, Leicester. LINCOLN.—Miss Sheppard (*pro tem.*), The County Hospital, Lincoln. LIVERPOOL.—Miss C. Worsley, Matron, Infirmary for Children. LONDON.—Miss Biggar, St. Thomas's Hospital, S.E. 1. MANCHESTER.—Miss Earl, Ancoats Hospital, Manchester. NORTHUMBERLAND AND DURHAM.—(1) Miss F. Jones, Royal Victoria Infirmary, Newcastle; (2) Miss M. Herbert, 3 St. Helen's Terrace, Low Fell, Newcastle. NOTTINGHAM.—Miss Taylor, Basford Sanatorium, Nottingham. PLYMOUTH.—Miss Bridge, Royal Albert Hospital, Devonport. SHEFFIELD.—Miss Hancox, Q.V.J.N.I., 334 Glossop Road, Sheffield. TRURO.—Mrs. Woodruffe, The Cottage, Probus, near Truro. YORKSHIRE CENTRE, AT LEEDS.—Miss Lindall, Women and Children's Hospital.

APPOINTMENTS BUREAU.

One of the most successful undertakings of the past few months has been the Appointments Bureau opened the week of the signing of the armistice, and organised to meet the needs of members seeking resettlement into civil life. A large number of applications have been dealt with, and some very good vacancies, both in the Colonies and at home, filled; and the Bureau serves another useful purpose in increasing the interest of members in the College and its work. Members from overseas especially express their pleasure in having an opportunity of visiting the College headquarters, and the comfortable and tastefully decorated club-rooms of the London centre.

PRESS.

The Council again expresses its gratitude to the nursing and general Press for the support it continues to give the College in keeping its activities before the profession and the public.

DEATHS.

It is with regret that the Council records the death of twenty-two members during the year. Five members have resigned.

DEMOBILISATION OF NURSES.

On behalf of nurses, the Council inquired into the question of the demobilisation of Army sisters at twenty-four hours' notice, and a letter was sent to the Secretary of the War Office and to the Director-General asking for their consideration of a suggestion to give members of the Army Nursing Services a month's full pay and allowances on demobilisation.

The Council recognised that there was no such provision in the agreement under which nurses entered the service, but it was pointed out that it would be a just and humane act if so many nurses who were broken in health, and all needing a holiday, were to receive a month's full pay, and allowances, so that a holiday could be taken free of financial anxiety, and the worry of looking for another post. This letter drew the reply that sisters in future would receive a week's notice on demobilisation, with one week's pay and allowances, and that the week in question might be taken as leave by sisters, should they so desire. This, though not entirely satisfactory, is an improvement on previous conditions.

Attention has also been drawn by the Council to the conditions of pay in the Royal Naval Nursing Service, where there has been no increase since the war, and where the scale is low and opportunities for promotion are limited. The Admiralty, in reply, stated that the subject was under consideration, and the Council awaits further developments.

REPRESENTATION ON ORGANISED BODIES.

The position of the College as a factor in nursing progress is now generally acknowledged, and during the last few months the College Council has been invited to appoint representatives to sit on—

(1) The Resettlement Committee, under the Ministry of Labour. Representatives, Miss Musson for England, and Miss Gill for Scotland on the Scotch Sub-Committee.

(2) The Subsidiary Health and Kindred Services Committee, under the Ministry of Reconstruction. Representative, Miss Clark.

(3) The Professional Women's Conference. Representative, Miss Coode.

(4) The Watching Council, under the Ministry of Health. Representative, Miss Cox Davies.

In this connection it will interest members to know that the following resolution was sent by the College Council to Dr. Addison, President of the Local Government Board:

"It is the opinion of the Council of the College of Nursing that on all Councils under the Ministry of Health dealing with questions of health trained nurses should be adequately represented."

The College is also affiliated to the National Council of Women of Great Britain and Ireland, and was represented on that Council last year by Miss Gill. On four Committees of the National Council the College is represented as follows:

(1) Parliamentary and Legislation Sectional Committee, Miss Cox Davies.

(2) Education Sectional Committee, Miss Lloyd Still.

(3) Mothercraft and Child Welfare Exhibitions Committee, Miss Peterkin.

(4) Public Health and Insurance Sectional Committee, Miss R. Henry.

ECONOMIC CONDITIONS IN THE NURSING PROFESSION.

The Council of the College has considered this question for some time, and has noted with pleasure that many hospitals have voluntarily raised the salaries of their nursing staffs. The Council recognises there is still need of reform in this matter, and has appointed a Committee to obtain particulars of the prevailing economic conditions in all branches of the profession. The Committee appointed is composed of persons in touch with all spheres of women's work, and is an *ad hoc* Committee, not including any members of the Council. Employers of nurses have been most helpful in placing valuable information at the disposal of the Committee, and nurses themselves were invited to lay before the Committee any facts or views they might have which bear upon the subject under inquiry.

ARTHUR STANLEY, *Chairman.*
M. S. RUNDLE, *Secretary.*

April 24, 1919.

**The College of Nursing Report** *(continued).*

### Fourth Annual Report of the Treasurers of the College.

The audited accounts as presented over the period from April 1, 1918, to March 31, 1919, during which time 5,047 members were registered and paid their fees, as against 5,414 in the previous year.

The surplus on the income and expenditure account for the year is £33,732 9s. 6d., which sum has been added to the accumulated fund of the College, and this fund now amounts to £42,425 0s. 4d.

Under the expenses of management there are three items, namely, salaries, £1,206 5s. 3d.; printing and stationery, £884 12s. 2d.; and postage, £444 9s., covering which a few details may be given.

The greater part of this expenditure was incurred in the registration department and in preparing a Register of the Members of the College. The expenses of this department must of necessity be heavy; thus the salaries amounted to £621 8s., the stationery and printing to £455 14s. 9d., and the postage to £300. During the year, from the registration department alone, 43,500 separate communications were despatched.

The legal expenses incurred with the proposed amalgamation with the Royal British Nurses' Association, the whole of which the College agreed to be responsible for, amounted to £269 0s. 10d.

During the year the Council appointed a Committee to inquire into the salaries and economic conditions prevailing in the nursing profession, and authorised a grant of £200 for the purpose. A first instalment of £50 has been made.

A grant of £70 was received from the British Women's Hospital Committee for scholarship fees, and of £32,500 from the Nation's Fund for Nurses.

The audited accounts of the Scottish and Irish Boards are submitted. Grants amounting to £785 9s. 3d. have been made during the year to these Boards.

The Council again offers its best thanks to Sir James Boyton for the loan of the offices at No. 6 Vere Street, free of all rent, rates, lighting, and heating.

The financial position of the College is most satisfactory.

(Signed) COMYNS BERKELEY,
SIDNEY BROWNE,
W. H. GOSCHEN,
Hon. Treasurers.

### ANNUAL REPORT OF THE SCOTTISH BOARD, 1918-19.

DURING the past year the Scottish Board has met four times, the Registration Committee eleven times, and the General Purposes Committee five times, the meetings being held alternately in Edinburgh and Glasgow.

The Board records with regret that during the year the following members resigned their seats on the Board, the reason being in each case that pressure of other work prevented them from taking an active part in the affairs of the College: Miss Gardner, Miss MacCalman, and the Rev. G. Montgomerie Campbell. In addition Miss Morison resigned on taking up professional work in England. As Miss Gardner and Miss MacCalman represented the members of the College who are in active nursing work, the Board nominated Miss Isabella Calder, 4th Scottish General Hospital, Glasgow, and Miss Isabel Bailie, 3rd Scottish General Hospital, Glasgow, to take their places. In the case of the other two members who resigned no nomination was made in view of the election of the new Scottish Board which is about to take place.

The development of the College in Scotland has made steady advance during the year. In all 510 applications for membership were adjudicated on by the Registration Committee. Of these 487 were passed, eleven rejected, three withdrawn, two referred to the Council of the College, and seven are still under consideration. The papers of 130 further candidates had not been fully dealt with at the end of the year. The number of members on the Scottish Register on March 31, 1919, was 1,288.

The Board felt strongly that in the unsettled state of affairs prevailing in the spring of 1918 it was inadvisable to proceed with the election of the Scottish Board, which ought to have taken place in the summer of last year, and the Council of the College adopted the recommendation of the Board that the election should be postponed till 1919. Arrangements have been made for the election taking place immediately after the 1919 election to the Council.

During the year the Board took up the question of the formation of Local Centres in different parts of Scotland, and being agreed as to the value of such Centres, drew up regulations for their constitution under the Board. As a consequence active Centres have been formed in Edinburgh and in Glasgow, and they have already been of great benefit in bringing the members of the College into touch with each other. Movements for the establishment of clubs for nurses in both these cities are now being proceeded with.

Propaganda work was carried on by the Secretary in Aberdeen and other places during the year.

In February 1919, the Board received with regret the resignation of Miss M. A. Brunton, the Secretary of the Board. They have elected Miss C. A. Pike in her place.

FINANCE.

The sum of £400 was received from the Honorary Treasurer of the College of Nursing for the year 1918, and the sum of £14 10s. 9d. was repaid to Council, being amount overpaid by them for year to March 31, 1918. The cash balance on hand on March 31, 1918, was £60 11s. 11d., and the expenditure for the year has been £386 13s. 3½d., leaving a cash balance on March 31, 1919, of £59 7s. 10½d.

JAMES RITCHIE, *Chairman.*
C. A. PIKE, *Secretary.*

### ANNUAL REPORT OF THE IRISH BOARD, 1918-19.

WHEN the last annual report of the Irish Board of the College was published, the outlook for the ensuing year, although cloudy, was not without hopeful gleams of sunshine. Reviewing the position at the close of that year, the Board are of opinion that on the whole the brighter gleams have prevailed. The disturbed state of the country and the violence of political feeling, extending into every sphere of life and work, have made propaganda necessarily limited and progress uphill. Nevertheless the Board are able to report that the number of applications received in the first three months of 1919 more than doubles the number received in the previous three months. The total membership of the College is now 13,014. Of these, the number of members trained in Ireland who have paid their registration fee is 521, while there are 266 members trained in England or Scotland, but working or having their homes in Ireland.

In connection with the Nation's Fund for Nurses, Ireland has contributed £10,000 since May of last year. The Nation's Tribute to Irish Nurses, inaugurated by Sir Arthur Stanley, owes its existence to the College of Nursing, but in consequence of the peculiar circumstances of this country the appeal was made by an independent Committee under the chairmanship of Lady Arnott, D.B.E., without any reference to the College. The money, earmarked for Ireland, is being administered here by a Committee representative of Dublin and the counties, and of the various nursing associations, including the College. There are already many applications for assistance, and, while it has not received the credit due to it, the College is gratified

**The College of Nursing Report** ***(continued)*****.**

by the knowledge that it was the means of establishing this much-needed fund for the relief of nurses in distress.

Since the last report was issued a reading- and rest-room has been opened for the use of College members at 23 Kildare Street, Dublin. The quiet comfort of this room and the up-to-date nursing literature have been much appreciated by nurses anxious to study. It was intended to hold a series of lectures, but, owing to the severity of the influenza epidemic, the project was postponed for a time.

In the last annual report it was stated that the Irish Board had collected statistics with reference to the salaries of nurses in hospital, institutional, and private work, and had determined that the improvement of the economic status of Irish nurses was one of the most urgent needs of this country. Working unobtrusively and perseveringly, the members of the Board have aimed steadily at the enlightenment of public opinion in this matter. Within the last few months the question has suddenly come very much to the front in Dublin. Several hospitals have been considering a revision of their nursing conditions, and the forward policy which the Irish Board adopted over a year ago is now being generally recognised as reasonable and urgent.

The Board have to report with deep regret the death of one of their most valued members, Dr. Charles Benson, F.R.C.S.I., whose interest in the College was unfailing, and who was a regular attendant at meetings. His loss is keenly felt by the Board, for he was greatly esteemed by every member.

In conclusion, the Board are glad to report that distinct progress has been made during the past year. The aims and objects of the College are becoming more generally known, and the realisation that the College stands for the highest in every department of nursing, in training, in educational improvement, in economic status, in professional development, and in public service, is attracting the best nurses, and surely, if slowly, building a firm stronghold in Ireland.

George Peacocke, Chairman.
Harriet E. Reed, Hon. Secretary.

### HOSPITAL AND INSTITUTIONAL RESULTS FOR 1918.

**The Royal Devon and Exeter Hospital, Exeter.** Since the foundation of the hospital in 1741 the number of in-patients treated has been 185,260. The published income and expenditure account includes many items which properly belong to a balance sheet, but apparently the receipts in 1918 were £15,732 and the expenditure £16,432.

**The Wolverhampton and Staffordshire General Hospital.**— The amount received last year from the Hospital Saturday Fund was £8,129, being an increase of £740 over 1917. The hospital had a surplus on the year of £912. A reconstruction scheme, including a new laundry and a new out-patient department, is being considered by the Board.

